# Effect of renal denervation on long-term outcomes in patients with resistant hypertension

**DOI:** 10.1186/s12933-021-01309-3

**Published:** 2021-06-05

**Authors:** Bo Liang, Yi Liang, Rui Li, Ning Gu

**Affiliations:** 1 Nanjing University of Chinese Medicine, Nanjing, China; 2 Southwest Medical University, Luzhou, China; 3 Nanjing Hospital of Chinese Medicine Affiliated to Nanjing University of Chinese Medicine, Nanjing, China

**Keywords:** Hypertension, Renal denervation, Resistant hypertension, Intervention

## Abstract

Increasing studies strongly prove that renal denervation, a minimally invasive surgery, is a promising new non-drug treatment method that can effectively control blood pressure in patients with resistant hypertension, but the evaluation of the long-term blood pressure control effect of renal denervation for resistant hypertension is still lacking. Here, we critically review current long-term follow-up data about the use of renal denervation for RH to comprehensively evaluate the effectiveness of renal denervation for RH, and to provide practical guidance for practitioners who are establishing a renal denervation service. Limited by the current research, many problems need to be solved before renal denervation is applied to RH. In addition, ambulatory blood pressure should be the first choice for the evaluation of blood pressure. Finally, the continuous antihypertensive effect of renal denervation in different renal denervation systems also needs to be strictly compared.

## Introduction

Resistance hypertension (RH) requires four or more antihypertensive agents to keep blood pressure (BP) below 130/80 mmHg [1], which accounted for about 25% [2, 3] among hypertension, a population health focus [4, 5]. RH is a growing clinical condition worldwide associated with target-organ damage and poor prognosis compared to non-RH [6, 7]. Renal denervation (RDN) therapy for RH has traveled a storied path over the past decade, from unbridled enthusiasm to abrupt disillusionment, and now renewed but still cautious interest [8]. While we review the earlier criticisms of trial conduct and more broadly device-based therapies for hypertension, we may need to understand the implications of these clinical trials based on RDN therapy more systematically and comprehensively. We previously summarized the typical clinical trials of RH treated with RDN in recent years and found that RDN therapy seems to be safe and reliable for RH although there are still many problems to be solved about this device-based therapy [9]. Here, we critically summarize the prospect of RDN’s role in clinical practice and seemingly validated the effects of RDN on long-term outcomes in patients with RH.

## Available evidence

**Fig. 1:**
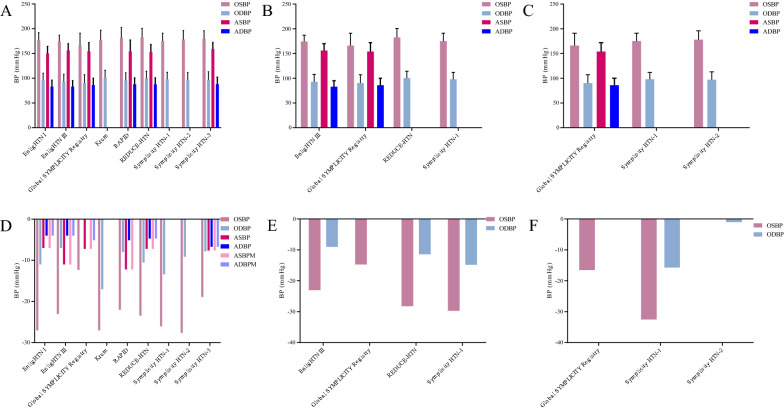
Effect of renal denervation on long-term outcomes in patients with resistant hypertension. **A**–**C** Baseline blood pressure at 12-, 24-, and 36-month. **D**–**F** Antihypertensive effect of RDN on blood pressure at 12-, 24-, and 36-month

We only included trials with a follow-up time of more than 12 months to reassess the effects of EDN on long-term outcomes in patients with RH. Based on our previous work [9], we included a total of nine typical clinical trials with a maximum follow-up of 3 years (EnligHTN I [10], EnligHTN III [11], Global SYMPLICITY Registry [12], Krum [13], RAPID [14], REDUCE-HTN [15], Symplicity HTN-1 [16], Symplicity HTN-2 [17], and Symplicity HTN-3 [18]) in this study. The baseline information is shown in Table 1; Fig. 1A–C.

**Table 1:** The baseline information of major trials

Trials	Country	Patients	Study design	Indicators	Follow-up (months)
EnligHTN I	Australia	46	First-in-human, prospective, multi-centre, non-randomized study	OBP and ABPM	12
EnligHTN III	Australia and New Zealand	39	First-in-human, prospective, multi-center, non-randomized study	OBP and ABPM	12, 24
Global SYMPLICITY Registry	Canada, Western Europe, Latin America, Eastern Europe, South Africa, Middle East, Asia, Australia, and New Zealand	2237	Prospective, open-label, single-arm, observational registry	OBP and ABPM	12, 24, 36
Krum	Australian and European	45	Single-arm	OBP	12
RAPID	Europe and New Zealand	50	Prospective, multicentre, single-arm study	OBP and ABPM	12
REDUCE-HTN	Germany	146	Prospective, multicentre, single-arm study	OBP and ABPM	12, 24
Symplicity HTN-1	Australia, Europe, and USA	153	Single-arm	OBP	12, 24, 36
Symplicity HTN-2	Australia	106	Randomized controlled crossovertrial	OBP	12, 36
Symplicity HTN-3	United States	535	Prospective, single-blind, crossover, randomized, sham-controlled trial	OBP and ABPM	12

Office BP (OBP), the most easily obtained indication in patients with hypertension, can directly and objectively evaluate the effect of BP reduction in those populations. All trials reported OBP. Notably, Global SYMPLICITY Registry [12] only reported office systolic BP (OSBP). During the 12-month follow-up, OSBP was reduced with a median range from 12.3 to 23.6 mmHg, while office diastolic BP (ODBP) was reduced with a median range from 7.0 to 13.4 mmHg (Fig. 1D). A total of four trials reported 24-month follow-up data. At 24 months, the antihypertensive effect of RDN was better than that of 12 months, regardless of OSBP and ODBP (Fig. 1E). At 36 months of follow-up, we obtained more encouraging results (Fig. 1F), except for Symplicity HTN-2 [17].

Ambulatory BP monitoring (ABPM) is recommended for the initial evaluation of all newly diagnosed persons with hypertension and evaluation of antihypertensive efficacy in BP trials [19]. However, among nine trials, only six trials reported ABPM. As OBP, ABPM can achieve better results with the extension of follow-up time (Fig. 1D).

## Limitations and future perspectives

Our results must be viewed in light of the limitations of this analysis. Differences in procedural aspects, postprocedural management, and follow-up protocol may have existed between the included trials. In addition, some trials are single-arm trials, so we lack a positive control or sham control. Thirdly, in order to evaluate the results of RDN on long-term follow-up, we have to omit some short-term follow-up trials, such as Symplicity HTN-Japan [20], DENERHTN [21], SPYRAL HTN-OFF MED [22], SPYRAL HTN-ON MED [23], RADIANCE-HTN SOLO [24], and SPYRAL HTN-OFF MED Pivotal [25]. Moreover, not all trials reported ABPM, which limits our assessment of the impact of RDN on ABPM. Finally, different RDN systems, such as OneShot™ ablation system [14], EnligHTN™ multi-electrode system [10, 26], second-generation EnligHTN™ system [11], and Vessix System [15, 27], were not investigated.

What else then is needed before accepting RDN as a safe and effective therapy for RH? The extent of RDN during the operation and the patency of renal nerve fibers at the later stage after ablation may be the next hurdle we need to overcome. Other important issues will need to be addressed in the future such as the effect of RDN on reducing the number of drugs (or drug dosages) for long-term BP control and hard endpoint data, such as cardiovascular outcomes. With the development of RDN, the continuous innovation of RDN technology and methods, the development about better evaluating the real-time success of RDN, and the improvement for identifying individuals who are most likely to benefit from RDN will ultimately determine whether RDN represents a feasible way to manage RH in the future.

Efferent (sympathetic) and afferent (sensory) fibers compose renal nerves, which coordinate renal function, central hemodynamics, and BP. Renal efferent nerves could increase renin secretion, promote tubular sodium reabsorption, and regulate renal blood flow [28], and these functions are proposed to underlie the antihypertensive effects of RDN [29, 30]. Experimentally, the stimulation of renal afferent fibers in animals elevates BP [31], and renal afferent nerve activity is increased in deoxycorticosterone-salt rats [32] and renovascular hypertensive mice [31]. In these models, dorsal rhizotomy or selective chemical ablation of renal afferent fibers reduces BP to the same extent as total renal denervation [31]. However, the mechanism responsible for the antihypertensive effect of RDN is still unclear. An elevated BP coupled to increased sympathetic drive [33, 34]. Specifically targeting afferent renal nerves in deoxycorticosterone acetate-salt model [32, 35, 36] or efferent nerves in Dahl salt-sensitive rats [37] chronically lower BP. More studies are needed to determine the importance of afferent or efferent fibers in the antihypertensive effect of RDN. However, current evidence does not support the dominant role of afferent or efferent nerves in the antihypertensive effect of RDN. A previous study points that global neurogenic pressor activity was significantly lower in BPH/2J mice 14 days after RDN, thereby, sympathetic control of either the renal vasculature (via efferents), or the nonrenal vasculature and heart (via afferents), are increased in hypertensive BPH/2J mice [33]. However, another study reported that global neurogenic pressor activity, assessed using the ganglion blocker pentolinium, was normal in BPH/2J mice after RDN [38]. That is to say, there is still a long way to go.

Moreover, the mechanism of RDN on RH needs to be further explored. A sustained fall in BP induced by RDN was associated with a reduction of intrarenal renin expression [38]. The lack of inhibition of the depressor effects of pentolinium and enalaprilat by RDN suggests that vasoconstrictor effects of the sympathetic nervous system or renin–angiotensin–aldosterone system are not involved [38]. Another study also confirmed RDN did not result in a significant change in endogenous catecholamines or renin–angiotensin–aldosterone system hormones at 6 months despite significant reductions in BP [39]. On the contrary, some hold that RDN is thought to interrupt the sympathetic-mediated neurohormonal pathway as part of its mechanism of action to reduce BP [40, 41]. In certain animal models, RDN demonstrated a reduction of renal tissue norepinephrine and plasma renin activity [42], a downregulation of the ACE/Ang II/AT1R axis, and upregulation of the ACE2/Ang-(1–7)/MasR axis in the kidney [43]. Clinically, these results are elusive and confounded. RDN noted a significant decrease in plasma renin activity [44, 45]. The greater the plasma renin activity, the more obvious the antihypertensive effect [45]. More studies indicated plasma renin activity did not significantly change post-RDN [46–48]. RDN also reduced whole-body norepinephrine spillover [49], did not significantly change plasma catecholamines [46], and had a mixed influence on aldosterone [45, 47, 48, 50]. In addition, arterial stiffness is a leading biomarker of risk in hypertension [51], and ferritin represents an independent risk factor of arterial stiffness [52]. However, the close relationship between ferritin and RH still needs further study.

## Conclusions

RDN may be one of the effective ways to reduce BP in patients with RH, but there are still many problems to be solved. The first is the nature of the study needs to be a double-blind, sham-controlled study. In addition, for the evaluation of BP, ambulatory BP needs to be the first choice. Finally, the continuous antihypertensive effect of RDN in different RDN systems also needs to be strictly compared. Still, we are seeing light at the end of the tunnel.

## Data Availability

Not applicable.

## Abbreviations

RH: Resistance hypertension
BP: Blood pressure
RDN: Renal denervation
OBP: Office blood pressure
OSBP: Office systolic blood pressure
ODBP: Office diastolic blood pressure
ABPM: Ambulatory blood pressure monitoring
